# The c-Myc/miR17-92/PTEN Axis Tunes PI3K Activity to Control Expression of Recombination Activating Genes in Early B Cell Development

**DOI:** 10.3389/fimmu.2018.02715

**Published:** 2018-11-22

**Authors:** David Benhamou, Verena Labi, Andrew Getahun, Eli Benchetrit, Reem Dowery, Klaus Rajewsky, John C. Cambier, Doron Melamed

**Affiliations:** ^1^Department of Immunology, Faculty of Medicine, Technion–Israel Institute of Technology, Haifa, Israel; ^2^Max Delbrück Center for Molecular Medicine, Berlin, Germany; ^3^Division of Developmental Immunology, Biocenter, Medical University of Innsbruck, Innsbruck, Austria; ^4^Department of Immunology and Microbiology, University of Colorado School of Medicine, Aurora, CO, United States

**Keywords:** B cell development, recombination activating gene (RAG), PI3K–AKT pathway, microRNA, PTEN (phosphatase and tensin homolog)

## Abstract

Appropriate PI3K signals generated by the antigen receptor are essential to promote B cell development. Regulation of recombination activating gene (RAG)-1 and RAG-2 expression is one key process that is mediated by PI3K to ensure developmental progression and selection. When PI3K signals are too high or too low, expression of RAGs does not turn off and B cell development is impaired or blocked. Yet, the mechanism which tunes PI3K activity to control RAG expression during B cell development in the bone marrow is unknown. Recently we showed that a c-Myc/miR17-92/PTEN axis regulates PI3K activity for positive and negative selection of immature B cells. Here, we show that the c-Myc/miR17-92/PTEN axis tunes PI3K activity to control the expression of RAGs in proB cells. Using different genetically engineered mouse models we show that impaired function of the c-Myc/miR17-92/PTEN axis alters the PI3K/Akt/Foxo1 pathway to result in dis-regulated expression of RAG and a block in B cell development. Studies using 38c-13 B lymphoma cells, where RAGs are constitutively expressed, suggest that this regulatory effect is mediated post-translationally through Foxo1.

## Introduction

The development of B lymphocytes in the bone marrow (BM) is a highly regulated process guided by the successive attempts to recombine immunoglobulin (Ig) genes and to assemble the B cell antigen receptor (BCR). Recombination activating gene (RAG)-1 and RAG-2 are key enzymes in this process and their expression is tightly regulated by signals generated by the antigen receptor to promote developmental progression through negative and positive selection check-points. Binding self-antigens activates tolerance mechanisms in immature and transitional B cells (1–3), whereas appropriate ligand-independent (tonic) signals are necessary for positive selection, developmental progression and maturation (4, 5). Thus, tonic signals, which are transmitted by the precursor BCR (preBCR), stimulate proliferative expansion of proB cells (6, 7), and those transmitted by the BCR promote developmental progression and maturation of immature B cells expressing non-harmful receptors (8–10). Failure to express preBCR or BCR or their signaling or surface regulatory components impairs or blocks B cell development (4, 11, 12).

The phosphoinositide 3-kinase (PI3K) activity is critical for B cell development and survival (12–16). Tonic PI3K signaling regulates expression of RAGs and is therefore essential for positive selection at the proB and immature B cell stages. Foxo1 is a major transcription factor necessary to induce the transcription of both RAGs. In developing B cells the expression and function of Foxo1 are directly regulated through phosphorylation and inactivation by the PI3K/Akt pathway (17, 18). When PI3K signals are too low, such as in CD19-deficient (CD19KO) B cells or when antigen receptor is ablated, continues RAG expression results in ongoing rearrangements (9, 14, 19–21). On the other hand, PI3K signals that are too high, such as in PTEN-ablated B cells, suppress RAG expression and impose a severe developmental block at the proB stage (22). These studies show that generation of appropriate PI3K activity is crucial to control RAG levels, thus allowing for proper developmental progression. However, the exact physiological mechanism and/or biochemical circuit which tunes PI3K activity to control RAG expression at these early stages of B cell development are unknown.

MicroRNAs were shown to regulate B lymphocyte fate at multiple levels, affecting physiological processes such as development, differentiation, and function (23–26). Recently, we showed that miRNAs are essential to regulate the PI3K/Akt/Foxo1 pathway and RAG expression in peripheral B cells (27). In another work, we reported an autostimulatory axis by which the PI3K regulates expression of its main antagonistic phosphatase, phosphatase and tensin homolog (PTEN). This autostimulatory axis is composed of a transcription factor c-Myc, a microRNA miR17-92, and a signaling molecule PTEN (c-Myc/miR17-92/PTEN axis) and controls PI3K activity for positive and negative selection of immature B cells (28). According this axis, c-Myc, whose expression levels are regulated by PI3K, directly controls expression of miR17-92, which post-transcriptionally promotes degradation of PTEN mRNA to dampen PTEN protein levels and to enhance PI3K activity. The resulting enhanced PI3K further promotes expression of high levels of c-Myc and survival of B cells (see also **Figure 8**). In the present study, we explored the role of the c-Myc/miR17-92/PTEN axis in regulating RAG expression at early stages of B cell development. We show that in proB cells the c-Myc/miR17-92/PTEN axis tunes expression of RAG through controlling the activity of PI3K. Using different conditional mutated mouse models we show that impaired function of the c-Myc/miR17-92/PTEN axis alters the PI3K/Akt/Foxo1 pathway to result in dis-regulated expression of RAG and a block in B cell development. In further studies we used 38c-13 B lymphoma cells, where RAG genes are constitutively expressed, to suggest that this regulatory effect is mediated post-translationally through Foxo1.

## Methods

### Mice

miR17-92^fl/fl^ (29), ROSA26STOPflox PTEN-2AYFP (PTENover) (30), ROSA26STOPflox-P110^*^ (16), CD19KO (31), miR17-92Tg mice (32), and mb1-cre (33) mice have been described. All mice are C57BL/6 or have been backcrossed to the C57BL/6 background for more than 10 generations. Mice were housed and bred at the animal facility of the Technion, Faculty of Medicine (Israel) or at the Max Delbrück Center for Molecular Medicine (Berlin, Germany) or at the University of Colorado, School of Medicine (Aurora CO, United States) and used for experiments at 10–12 weeks of age. Mouse studies at the Technion were approved by the local committee for the supervision of animal experiments. Animal care in Berlin followed guidelines of the Max Delbrück Center for Molecular Medicine and the governmental review board (Landesamt für Gesundheit und Soziales Berlin, LaGeSo). Animal studies at University of Colorado were performed in accordance with the regulations and with approval of the University of Colorado Denver Institutional Animal Care and Use Committee.

### Cells and transfection

38c13 cells (34) were cultured in standard RPMI. Cells were infected with different viral vectors to express a sponge system to miR-19b or to the complete miR17-92 cluster, overexpress PTEN or knockdown PTEN (shPTEN) (see supplemental details). Stable transfectants were selected and used for the experiments. Cells were lysed (see Supplemental Details in methodology) for western blotting and quantification of mRNAs. In some experiments a specific inhibitor for Foxo1 transcription activity AS1842856 (Calbiochem) was added to the cultured cells at 14 μM for 2 h. In some experiments HEK cells were transfected with vectors encoding luciferase reporter bound to 3′UTR of PTEN or Foxo1 as detailed in the Supplementary Section. IL-7-driven BM cultures for B cell precursors were prepared as previously described (35). Cells grown in these primary cultures were harvested after 5 days (>95% B220+) and were used as detailed. In some experiments B cell precursors were grown in IL-7 driven BM cultures (36) and cells were treated with 1 μM miR19b antagomirs (Exiqon).

### Flow cytometry and cell sorting

Single cell suspensions from mouse BM or spleen were stained with fluorescently labeled antibodies for surface and intracellular proteins to identify specific B cell subsets. Antibodies for the following surface markers were used: αB220-BV785 (RA3-6B2), αCD19-BV605 (6D5), αCD93-APC (AA4.1), αB220-PB (RA3-6B2), αCD25-PE (3C7), αc-kit-BV605 (ACK2) from BioLegend, αCD1d-PE (1B1) from eBioscience. Antibodies for intracellular staining used were anti pAkt (4060) and anti PTEN (p559) from Cell Signaling using the BrdU fixation kit (552598) from BD. Data were acquired on LSRFortessa (BD Pharmingen) and analyzed using FlowJo software (Tree Star). In some experiments cells of specific populations based on surface markers expression were sorted using FACSAria (BD).

### Western blot analysis

Western blots were performed as we have previously described (37). Briefly, cells were lysed in RIPA buffer containing complete protease inhibitor cocktail (Roche Diagnostics), 1 mM sodium orthovanadate, 5 mM sodium fluoride and 0.5 mM phenylmethylsulfonyl fluoride. Proteins were separated by SDS-PAGE and transferred to polyvinylidene fluoride membrane (Millipore, Bedford, MA, United States). Blots were probed with rabbit anti-phospho-Akt (Ser473) (Cat 4060), rabbit anti-Akt (Cat 9272), rabbit anti-PTEN (Cat 9188), (all from Cell Signaling), rabbit anti-Foxo1(Santa Cruz), mouse anti-actin (C4) (from MP biomediacls). To reveal bound Abs we used HRP conjugated secondary Abs. Blots were developed with ECL reagent (Pierce).

### Viral vectors

To suppress miR19b or the entire miR17-92 cluster in 38c13 cells we used pMSCV-PIG-sp19 or pMSCV-PIG-sp17-92 encoding a sponge system to miR19b or to the entire miR17-92 cluster as has been described (38). To overexpress human PTEN we used an inducible Tet-on expression system that has been described (28). PTEN expression was induced upon induction with 1 μg/ml doxycycline. To knockdown PTEN expression we used a viral vector encoding shPTEN [LENG.ShPten 1523, (39)]. In some experiments, 38c13 cells were first infected with LENG.ShPten1523 vector and after selection the cells were infected with pMSCV-PIG-sp19 to generate cells where PTEN is knockdown and miR19b is suppressed.

### Viral packaging and infection

Retroviral infections, virus production infection and selection of cells were performed as we have described (28, 40).

### Quantitative PCR

Quantitative PCR analysis for RAG-1 RAG-2, PTEN and Hprt were performed as we have previously described (40). Briefly, RNA was extracted by using Tri-Reagent (Sigma) and reverse transcribed to cDNA (Promega). Real-time PCR was performed using Syber green mix (Tiangen) using BioRad CFX connect Real-Time PCR. Samples were normalized with Hprt gene amplification. PCR product specificity was confirmed by melting curve analysis where the crossover (Ct) values were used to calculate gene specific input mRNA amount for each sample according to the calibration curve method. DATA were analyzed using the CFX Manager Software.

### Luciferase assay

To perform the luciferase reporter assay, 10^5^ HEK cells per well in 1 ml of medium were seeded in 24-well plates for 24 h. Cells were then transfected with the modified firefly luciferase vector PmiRGLO- PTEN3'UTR or the PmirGLO-Foxo13′UTR together with a vector encoding miR17-92 or GFP using Calfectin according to the manufacturer's instruction. The 3'UTRs of PTEN and Foxo1 were amplified from DNA isolated from C57BL/6 mice by pcr and cloned into the pmirGLO vector. After 48 h the firefly and renilla luciferase activities were measured with the dual-luciferase reporter assay system (Promega). To control the transfection efficiency, firefly activity was normalized to renilla activity.

### Statistical analysis

The statistical significance of the differences between experimental groups was determined using unpaired two-tailed Student's t test with differences considered significant at *p* < 0.05.

## Results

### Ablation of miR17-92 in ProB cells imposes a mild pro-to-pre B cell block with elevated expression of RAGs

**Figure 1:**
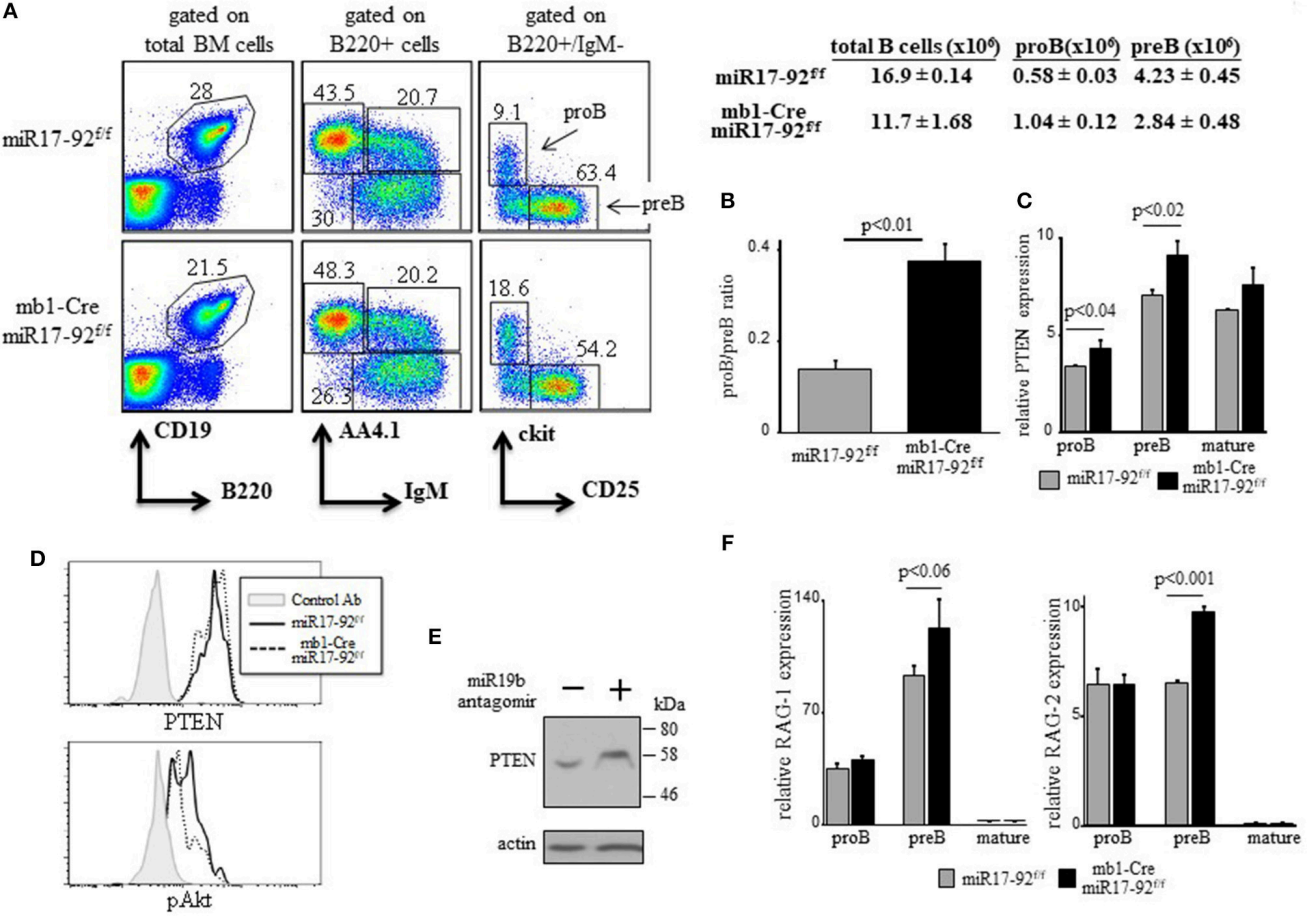
Ablation of miR17-92 in proB cells imposes a mild pro-to-pre B cell block and elevates expression of RAGs. **(A)** Representative flow cytometry analysis of BM cells from mice with the indicated genotypes (3 mice from each genotype). Initial forward and side scatter gates were set to exclude dead cells and debris. Numbers adjacent to outlined areas indicate % cells amongst total BM cells in each gate. The proB (B220 + IgM- AA4.1 + CD25- ckit+) and preB (B220 + IgM- AA4.1 + CD25 + ckit-) populations are marked with arrows. Also shown are absolute cell counts. **(B)** The proB and preB cells were quantified for each individual mouse and are expressed as proB/preB ratio. Plot depicts mean from 3 individual mice ± SE. **(C)** The proB, preB and mature B (B220 + IgM + AA4.1-) cells were sorted from the gates shown in **(A)** and analyzed for relative expression of PTEN mRNA by qPCR and normalized to Hprt. Results are presented as mean from 3 individual mice ± SE. **(D)** Intracellular stain for PTEN and pAKT of BM cells gated on B220+/IgM- pro/pre B cells. Graph represents 2 mice in each group. **(E)** BM culture wild-type pro/preB cells were treated with or without miR19b antagomirs for 48 h and analyzed for the indicated proteins by western blotting. **(F)** Sorted proB, preB and mature B cells were analyzed for relative expression of RAG-1 (top) and RAG-2 (bottom) by qPCR normalized to Hprt. Graph depicts mean from 3 individual mice ± SE.

We have shown that a c-Myc/miR17-92/PTEN axis regulates PI3K activity for positive selection of immature B cells (28). As PI3K signals are necessary to turn off RAG (17, 18, 20), we tested whether the c-Myc/miR17-92/PTEN functions to tune PI3K activity to control RAG expression during B cell development. To do so *in vivo*, we used conditional mice enabling altering the expression and/or activity of c-Myc/miR17-92/PTEN axis in B lineage cells. We first generated mb1-Cre/miR17-92^f/f^ mice, where the entire miR17-92 cluster is conditionally ablated in early proB cells (33). Analysis of BM cells in these mice revealed a mild block at the proB stage as reflected by a 2-fold increase in their frequency (Figure 1A) and by the changed proB/preB cell ratio (Figure 1B). In agreement with Lai et al (41), we found that ablation of miR17-92 resulted in 15–20% increase in expression of PTEN mRNA in proB and preB cells (Figure 1C) and PTEN protein (Figure 1D), and reduced PI3K activity measured by Akt phosphorylation (Figure 1D). These changes were also validated in pro/pre B cells grown in BM cultures that were treated with miR19b antagomirs (Figure 1E). To further validate these findings in a reciprocal experiment we analyzed hCD2Cre/R26miR17-92stop^flox^ mice overexpressing miR17-92 in all lymphocytes and found that PTEN expression is reduced in pro/pre B cells whereas pAkt is increased (Supplementary Figure 1B). Consistent with this and with our hypothesis, we found that expression levels of both RAG-1 and RAG-2 were elevated in preB cells from mb1-Cre/miR17-92^f/f^ mice relative to the controls (Figure 1F). Despite of this mild pro-to-pre B cell block we found no significant differences in splenic B cells (total number and specific subsets, Supplementary Figure 1). These findings suggest that intrinsic deletion of miR17-92 in proB cells impairs the regulatory activity of the c-Myc/miR17-92/PTEN axis to result in enhanced RAG expression and a partial pro-to-preB block.

### PTEN overexpression partially blocks pro-to-pre B cell transition and elevates expression of RAGs

**Figure 2:**
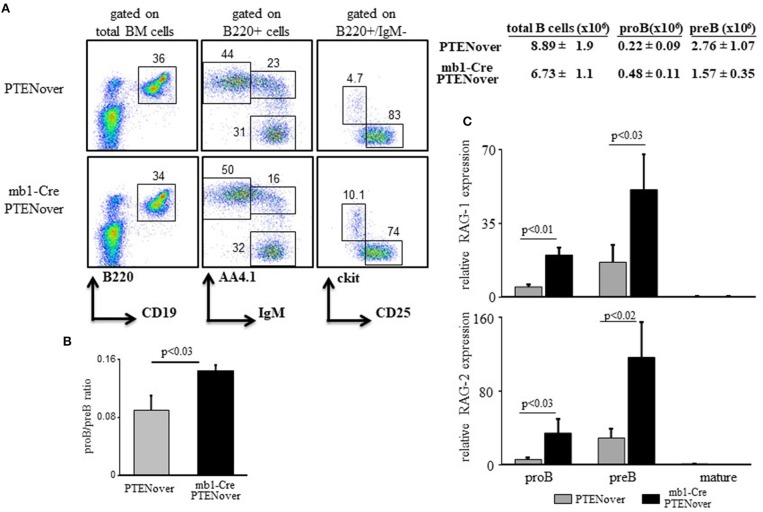
PTEN overexpression partially blocks pro-to-pre B cell transition and elevates expression of RAGs. **(A)** Representative flow cytometry analysis of BM cells from mice with the indicated genotypes (3–5 mice from each genotype). Initial forward and side scatter gates were set to exclude dead cells and debris. Numbers adjacent to outlined areas indicate % cells amongst total splenocytes in each gate. Also shown are absolute cell counts. Total numbers of B cells in the BM were calculated as total number of nucleated cells purified from one femur and one tibia multiplied by percentage of B220+ cells. **(B)** The proB and preB cells (gated as in Figure 1 legend) were quantified for each individual mouse and are expressed as proB/preB ratio. Plot depicts mean from 3 to 5 individual mice ± SE. **(C)** The proB, preB, and mature B cells were sorted using the indicated gates and analyzed for relative expression of RAG-1 (top) and RAG-2 (bottom) by qPCR normalized to Hprt. Graph depicts mean from 3 to 5 individual mice ± SE.

To confirm the function of the c-Myc/miR17-92/PTEN axis in tuning RAG expression in early B cell development we generated mb1-Cre/ROSA26STOPflox PTEN-2AYFP compound mice (30), where selective over-expression of PTEN by about 15–20% is obtained in the B lineage starting from the proB stage (Supplementary Figure 1C). Over-expression of PTEN in these mice is encoded by PTEN cDNA and is therefore not subjected to regulation by the miR17-92. Analysis of B cell development and RAG expression in these compound mice recapitulated our findings obtained in the miR17-92 ablated mice (Figure 1). Thus, overexpression of PTEN imposed a partial block at the proB stage as revealed by about 2-fold increase in proB cell frequency (Figure 2A) and a changed proB/preB cell ratio (Figure 2B). Similarly, overexpression of PTEN, obtained by transduction of hematopoietic stem cells (HSCs) with a lentivirus vector encoding human PTEN, impaired B cell development as reflected by reduced frequencies of total B cells in BM and spleen, and accumulation of B220+/CD43 + cells (mostly proB cells). However, when the transducing vector encoded a human PTEN construct ligated to a miR19b binding site [the major miRNA regulating PTEN (32)] PTEN expression was suppressed and B cell development was rescued (Supplementary Figure 2). Quantification of RAG expression in sorted cells revealed that RAG-1 and RAG-2 were significantly increased in proB and preB cells that over-express PTEN (Figure 2C). These results suggest that the autostimulatory c-Myc/miR17-92/PTEN axis tunes RAG expression in B cell development through balancing PI3K activity. Alteration of this axis, by either ablation of the miR17-92 cluster or over-expression of PTEN, enhances expression of RAG and partially blocks B cell development at the proB stage.

### The c-Myc/miR17-92/PTEN axis balances PI3K activity to control RAG expression in 38c13 cells

**Figure 3:**
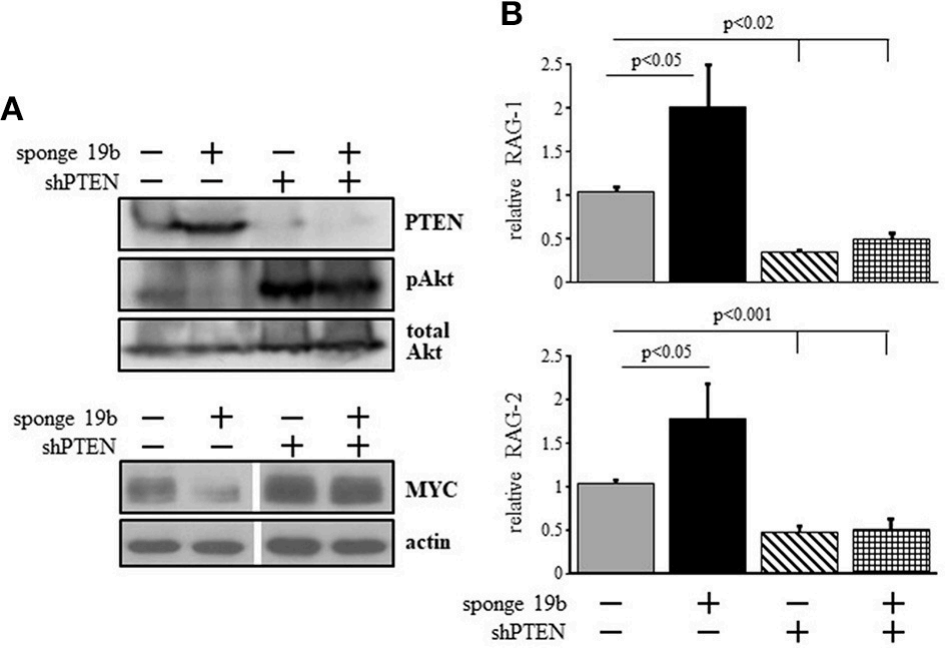
The c-Myc/miR17-92/PTEN axis regulates RAG expression in 38c13 cells through balancing PI3K activity. 38c13 cells were infected with vector encoding miR19b sponge or shPTEN (to suppress miR19b or PTEN, respectively) or with both, and stable transfectants were selected. **(A)** Representative western blot for the indicated proteins (4 different experiments). **(B)** The indicated stably transfected 38c13 cells were lyzed and analyzed for relative RAG-1 and RAG-2 expression by qPCR normalized to Hprt. Graph depicts mean of 4 experiments ± SE.

To probe for the mechanism by which the c-Myc/miR17-92/PTEN axis controls RAG expression we used the 38c13 immature B lymphoma cells which constitutively express RAG (34). To alter c-Myc/miR17-92/PTEN axis in these cells we used a sponge system specific to miR19b (38) to suppress expression of this miRNA. We found that suppression of miR19b resulted in a significant increase in expression of PTEN protein and a consequential reduction in PI3K activity as revealed by Akt phosphorylation (Figure 3A). Consistent with the reduced PI3K activity, expression of RAG-1 and RAG-2 were significantly enhanced by ~2-fold relative to the control (Figure 3B). Reciprocally, transfection with a vector encoding PTEN specific shRNA [shPTEN (39)] enhanced Akt phosphorylation significantly and suppressed expression of RAG-1 and RAG-2 by ~2-fold relative to the control (Figures 3A,B). Furthermore, in cells co-transfected with miR19b-sponge and shPTEN, activity of PI3K was enhanced to effectively suppress RAG expression (Figures 3A,B), suggesting that the increased expression of RAG induced by the miR19b-sponge system is mediated via alteration of PTEN expression. Consistent with the regulatory feedback of the c-Myc/miR17-92/PTEN axis we show that reduced PI3K activity obtained by suppression of miR19b reduced expression of MYC whereas enhanced PI3K activity obtained by suppression of PTEN enhanced MYC expression (Figure 3A).

**Figure 4:**
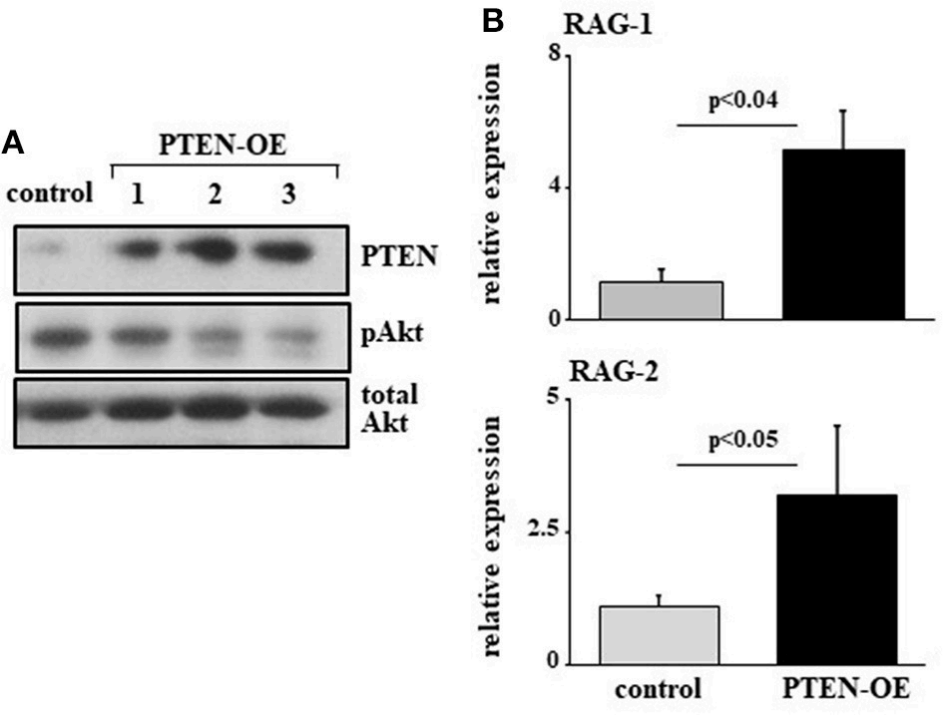
Over expression of PTEN suppresses PI3K and enhances RAG expression in 38c13 cells. 38c13 cells were stably transfected with a Tet-on inducible system to over-express human PTEN (PTEN-OE). Cells were treated with doxycycline and analyzed after 24 h. **(A)** Representative western blot for the indicated proteins (shown are three different clones for PTEN-OE). **(B)** The stably PTEN-OE and control 38c13 cells were lyzed and analyzed for relative RAG-1 and RAG-2 expression by qPCR normalized to Hprt. Graph depicts mean of 3–6 experiments ± SE.

To further validate this, we infected 38c13 cells with a vector encoding an inducible system for human PTEN expression [PTEN-OE (42)]. Over expression of PTEN in these cells was evident by PTEN protein level and a suppressed PI3K activity (revealed by Akt phosphorylation) in the selected subclones (Figure 4A). In agreement with the results obtained upon introducing the miR19b-sponge system (Figure 3), we found that RAG-1 and RAG-2 were significantly elevated in 38c13 cells over-expressing PTEN (Figure 4B). Collectively, these results confirmed that in 38c13 cells the regulation of RAG expression is mediated by the c-Myc/miR17-92/PTEN axis.

### Regulation of RAG expression by the C-Myc/miR17-92/PTEN axis is mediated post-translationally through Foxo1, but not post-transcriptionally through direct binding of miR17-92 to the Foxo1 3′UTR

**Figure 5:**
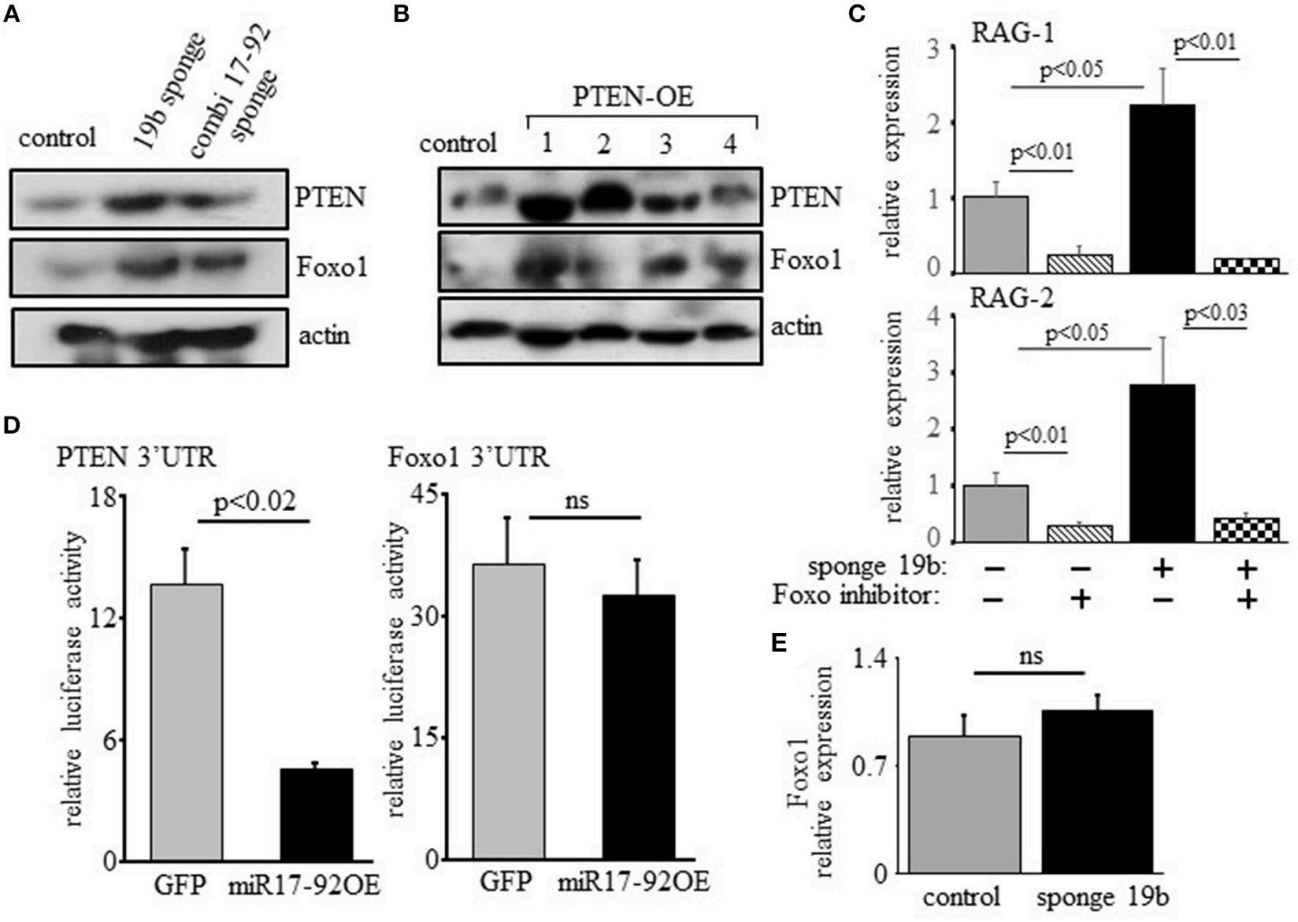
Regulation of RAG expression by the c-Myc/miR17-92/PTEN axis is mediated through Foxo1, but not through direct binding of miR17-92 to the Foxo1 3'UTR. **(A)** 38c13 cells were transfected with a sponge system to miR19b or to the entire miR17–92 cluster. Shown is a representative western blot for the indicated proteins (from 3 experiments). **(B)** 38c13 cells were stably transfected with a Tet-on inducible system to over-express human PTEN (PTEN-OE). Cells were treated with doxycycline and analyzed after 24 h lysed and analyzed by western blotting for the indicated proteins (shown are four different clones for PTEN-OE). **(C)** 38c13 cells that are wild-type or transfected with the miR19b sponge system were cultured for 2 h in the presence of Foxo1 inhibitor. Cells were then lyzed and analyzed for relative RAG-1 and RAG-2 expression by qPCR normalized to Hprt. Graph depicts mean of 3 experiments ± SE. **(D)** HEK cells were transfected with vector encoding luciferase reporting system that is ligated to PTEN3′UTR or to Foxo1 3'UTR, together with a vector encoding miR17-92 or GFP. After 48 h luciferase activity was determined. Graph depicts mean of 3 experiments ± SE. **(E)** 38-c13 cells that are wild-type control or transfected with the miR19b sponge system were lyzed and relative Foxo1 mRNA was quantified by qPCR normalized to Hprt. Graph depicts mean of 3 experiments ± SE.

Previous studies have shown that regulation of RAG by PI3K is mediated post-translationally through phosphorylation and inactivation of Foxo1 (17, 18). In agreement with this we find elevated expression of Foxo1 in 38c13 cells transfected with miR17-92 or the miR19b sponge (Figure 5A), or over-expressing PTEN (Figure 5). Moreover, we show that the induction of RAG expression obtained upon transfection with the miR19b sponge system is blocked by a Foxo inhibitor (Figure 5C), suggesting that the regulatory effects of the c-Myc/miR17-92/PTEN axis on RAG expression are mediated by Foxo1. Yet, bioinformatic analysis of the Foxo1 3′UTR (using the TargetScanHuman program; http://www.targetscan.org) revealed a potential binding site for miR19b, proposing a direct post-transcriptional regulatory effect of miR19b on expression of Foxo1. To test this we transfected human embryonic kidney (HEK) cells with a vector encoding luciferase reporter ligated to Foxo1 3′UTR or PTEN 3'UTR, together with a vector encoding miR17-92 or GFP control. The results in Figure 5D show that miR17-92 significantly reduced luciferase activity in HEK cells transfected with a luciferase reporter ligated to PTEN 3'UTR, whereas no change was recorded in HEK cells transfected with luciferase reporter ligated to Foxo1 3′UTR. Moreover, quantification of Foxo1 mRNA in 38c13 cells transfected with miR19b sponge revealed no significant change relative to control cells (Figure 5E). These results suggest that regulation of Foxo1 expression by the c-Myc/miR17-92/PTEN axis is mediated post-translationally through balancing PI3K activity.

### Overexpression of miR17-92 suppressed RAG expression in developing CD19KO B cells

**Figure 6:**
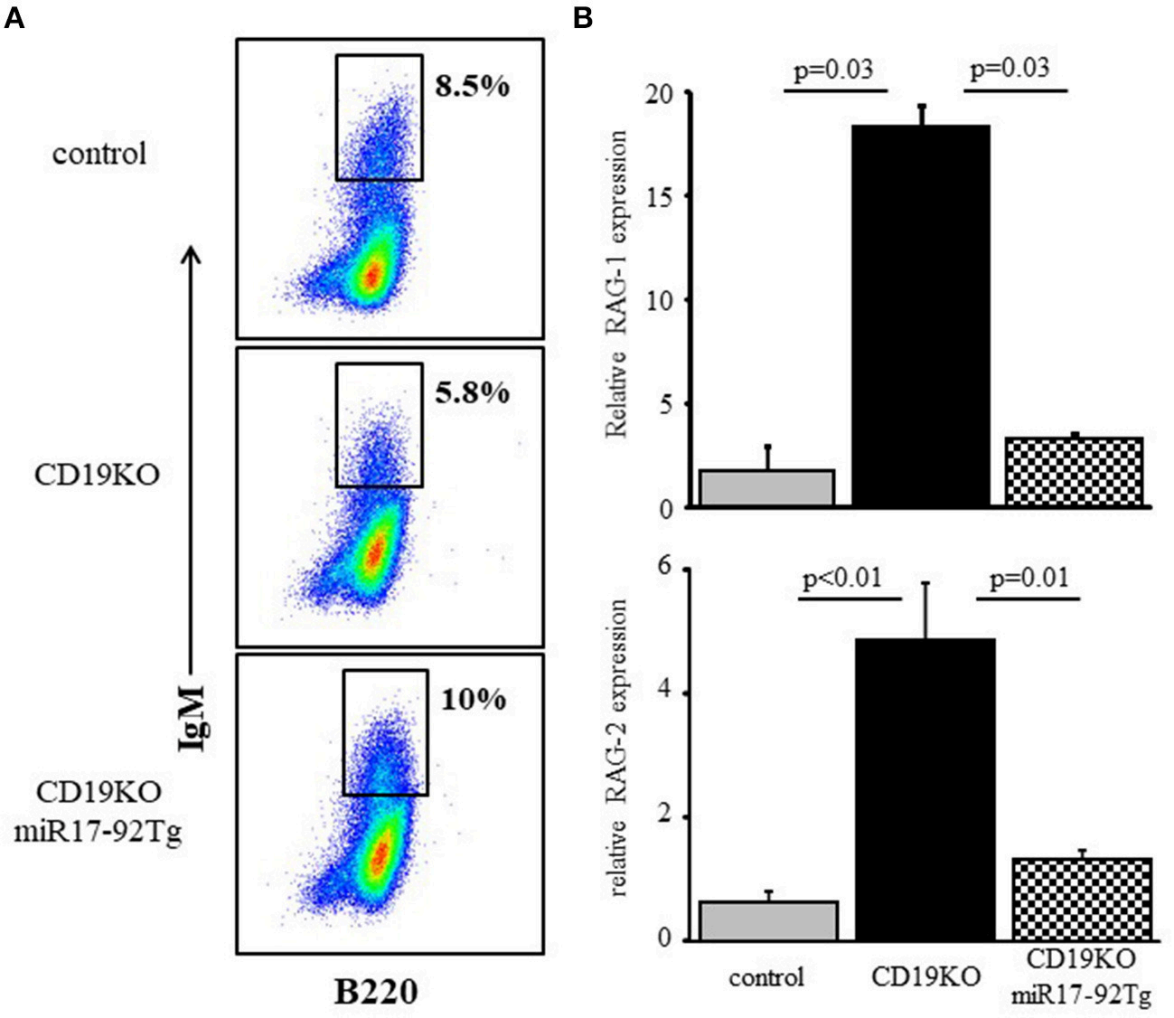
Overexpression of miR17-92 suppresses RAG in developing CD19KO B cells. IL-7-driven BM cultures from mice of the indicated genotypes were prepared. **(A)** Cells grown were harvested on day 5, stained for the indicated surface markers and analyzed by FACS. Initial forward and side scatter gates were set to exclude dead cells and debris. Shown are representative plots from 3 to 4 mice from each group. **(B)** Cells were lyzed and relative RAG-1 and RAG-2 mRNA was quantified by qPCR normalized to Hprt. Graph depicts mean of 3–4 mice from each group ± SE.

To address physiological roles of the c-Myc/miR17-92/PTEN axis in controlling RAG expression during B cell development we used CD19KO mice, where RAG expression in developing B cells does not turn off (9) owing to impaired PI3K signaling (20, 43). Consequentially, B cell maturation in CD19KO mice is impaired with evidence for ongoing Ig gene rearrangements (9, 19, 44). Overexpression of miR17-92 (28) or loss of PTEN (45) in developing CD19KO B cells enhanced PI3K activity, to effectively reconstitute maturation and differentiation of CD19KO B cell (28). The results in Figure 6 show that enhancing PI3K activity through overexpression of miR17-92 effectively suppressed RAG expression in developing CD19KO miR17-92Tg B cells to promote their differentiation in BM cultures. Thus, while RAG-1 and RAG-2 expression were significantly elevated in developing CD19KO B cells (~18 and ~5-fold, respectively), expression of both RAGs in developing CD19KO miR17-92Tg B cells was suppressed to a level that is not different from that found in developing control B cells. These results suggest that reconstitution of B cell development and maturation in CD19KO miR17-92Tg mice is mediated by the suppression of RAG expression, which is in turn mediated through tuning PI3K activity by the c-Myc/miR17-92/PTEN axis.

### Enhanced PI3K activity abolishes RAG expression and blocks B cell development at the early proB stage

**Figure 7:**
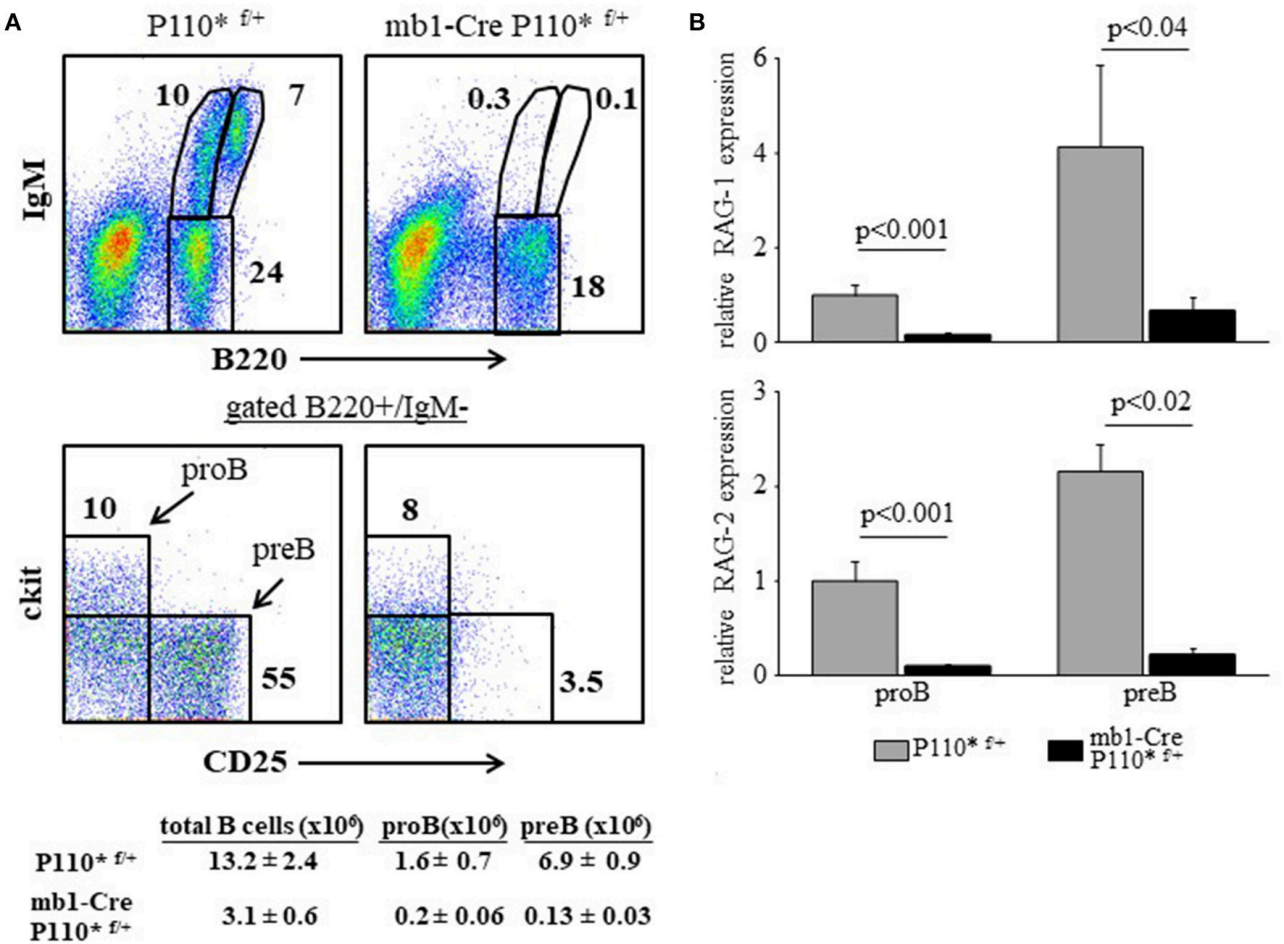
Enhanced PI3K activity abolishes RAG expression and blocks B cell development at the early proB stage. **(A)** Representative flow cytometry analysis of BM cells from mice with the indicated genotypes (3 mice from each genotype). Initial forward and side scatter gates were set to exclude dead cells and debris. Also shown are absolute cell counts. **(B)** The proB and preB cells were sorted using the indicated gates and analyzed for relative expression of RAG-1 (top) and RAG-2 (bottom) by qPCR normalized to Hprt. Graph depicts mean from 3 individual mice ± SE.

In all mouse models detailed here earlier (miR17-92 ablated, PTEN-over expression and CD19KO) B cell development is perturbed due to a low PI3K activity and enhanced RAG expression, resulting from reduced activity of the c-Myc/miR17-92/PTEN axis. To further confirm the importance of this axis we performed a reciprocal experiment and tested whether constitutively active PI3K would suppress RAG gene expression in developing B cells to impair their development. To do so, we generated mice carrying a conditional allele encoding constitutively active PI3K subunit P110α [P110^*^ (16)] specifically expressed in the B lineage starting at the early proB stage (mb1-Cre/ROSA26STOPflox-P110^*^ compound mice). As shown in Figure 7, the enhanced PI3K activity blocked B cell development at the early proB stage (ckit-/CD25-) with only few proB (cKit+/CD25-) and preB cells (cKit-/CD25+) detected (Figure 7A), explaining the nearly complete absence of B220+/IgM+ cells in the BM (Figure 7A). Analysis of RAG in sorted proB and preB cells from mb1-Cre/ROSA26STOPflox-P110^*^ mice revealed a significantly suppressed expression in both B cell subsets relative to control (Figure 7B). These results suggest that enhanced PI3K activity suppresses RAG expression to block B lymphopoiesis.

## Discussion

This study suggests that the autostimulatory c-Myc/miR17-92/PTEN axis functions as a post-translational mechanism balancing the PI3K/Foxo1/RAG pathway during early stages of B cell development. We show that modification of this axis altered expression of RAGs and impacted B cell development. Hence, while BCR-mediated PI3K signaling is most important for B cell development and survival, the c-Myc/miR17-92/PTEN axis functions to properly tune it for regulation of RAGs and to promote developmental progression.

**Figure 8:**
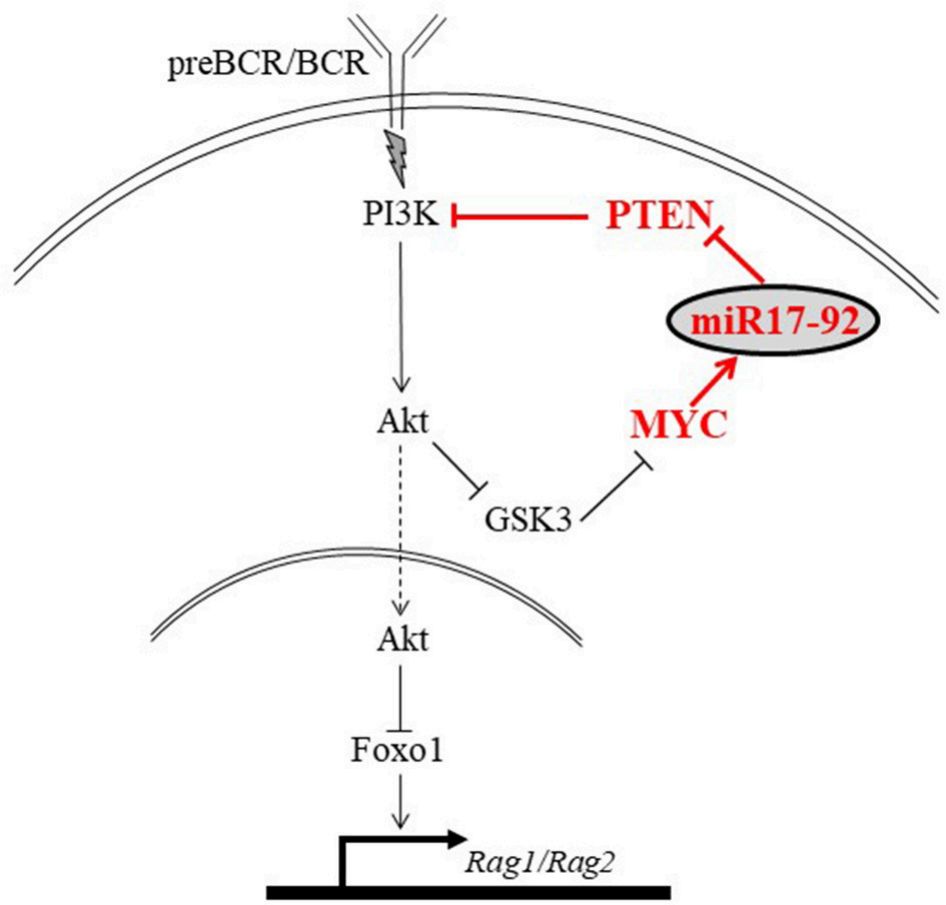
An illustration proposing a mechanism by which the MYC/miR17-92/PTEN axis balances the PI3K/Foxo1/RAG pathway in developing B cells. Signals generated by the antigen receptor activate PI3K/Akt to phosphorylate and inactivate Foxo1, leading to suppression of RAG. We propose that in developing B cells PI3K activation is balanced by the autostimulatory MYC/miR17-92/PTEN axis (shown in red). Accordingly, c-Myc regulates expression of miR-17-92 which reduces expression of PTEN to enhance PI3K activity and promote suppression of RAG.

The mechanism by which the c-Myc/miR17-92/PTEN axis balances the PI3K/Foxo1/RAG pathway in B cell development is illustrated in Figure 8. In developing B cells the PI3K/Akt/Foxo1 is most important to control RAG expression. Both, enhanced or suppressed PI3K significantly impact RAG expression and consequentially alter B cell development. Thus, enhanced PI3K activity obtained upon ablation of PTEN suppresses RAG expression and blocks B cell development at the proB stage (22), whereas deficiency of CD19 (9), or lack of the p85(alpha) regulatory subunit of PI3K (20) suppresses PI3K activity to result in ongoing RAG expression and Ig gene rearrangements (9, 19, 44). This study shows that the PI3K activity in developing B cells is balanced by the c-Myc/miR17-92/PTEN axis for appropriately controlled RAG expression (Figure 8). Accordingly, we show that altering the c-Myc/miR17-92/PTEN axis, by deletion of miR17-92 or by overexpressing PTEN, suppressed PI3K activity, led to enhanced RAG expression in early developing B cells, and imposed a mild pre-to-pro B cell block (Figures 1, 2). A similar mild pro-to-pre B cell block is also evident in CD19-/- mice, although RAG expression levels in pro and pre B cells were not measured in this study (46). Yet, in all these mutated mouse models significant B cell development is still observed, as revealed by the number of mature B cells in BM and spleen, probably suggesting the existence of compensatory mechanisms to enhance PI3K activity (9, 44). In agreement with this, Sandoval et al reported that cycling large preB cells express MYC and high level of inactivated (phosphorylated) Foxo1, whereas non-cycling preB cells do not have MYC protein but have abundant expression of RAG and DNA rearrangements (47).

On the other hand, it is more difficult to compensate for enhanced PI3K activity as B cell development in PTEN-ablated mice or transgenic for P110^*^ is blocked at the proB or early proB stage [respectively, (22) and Figure 7]. Owing to the increased PI3K activity and the resulting enhanced phosphorylation and degradation of Foxo1, RAG expression in pro and pre B cells from these mice was too low and probably insufficient to promote Ig gene rearrangements. This loss of Foxo1 also impairs expression of the transcription factor Ikaros that is necessary for Ig gene rearrangements (22). Remarkably, in the P110^*^ mice B cell development is blocked at an earlier stage relative to that observed in RAG-deficient mice (pre-proB in P110 relative to proB in RAG-/-), suggesting that in addition to the suppression of RAG other mechanisms may be involved. Studies have shown that developmental progression from pre-proB to the preB stage is mediated through transcriptional regulation by EBF or E2A (48), as well as by expression and signaling of the proBCR (8, 49). Hence, it is possible that the overly activated PI3K in the P110^*^ mice impairs expression and/or function of EBF and E2A, or alters expression and signaling of the proBCR to abort B cell development at the pre-proB stage. In addition, transcription from the RAG locus is required for transition into the earliest lymphoid progenitor stage (50). In RAG–/– mice this transcription allows differentiation to proB cells but not beyond due to the non-functional RAG mutation. It is possible that the overly activated PI3K blocks this transcription and prevents development of pre-proB to a proB stage in the P110^*^ mice.

In mature B cells a PI3K/Akt/Foxo1 pathway has been shown to control survival (16). Interestingly, suppressing PI3K by chemical inhibitors or by ablating biogenesis of miRNAs stimulates expression of RAG in mature splenic B cells (27). As tonic PI3K activity increases with B cell maturation to promote survival (3), it is possible that the increased PI3K activity is also important to prevent re-expression of RAGs in peripheral B cells. Thus, during B cell development in the BM the c-Myc/miR17-92/PTEN axis regulates PI3K for RAG expression (this study) and for positive and negative selection of immature B cells (28), but the regulatory circuits balancing tonic PI3K activity for survival and to seal-off RAG expression are less known.

The enhanced expression of RAG in developing B cells was delayed to some extent in miR17-92-ablated mice relative to PTEN over expressing mice. Thus, while differences in RAGs were detected only in preB cells in miR17-92-ablated mice, significant elevation in RAGs was readily seen already in proB cells in PTEN over expressing mice. We attribute this to the elapsing time from genomic deletion until actual affecting PI3K activity. Thus, in PTEN over expressing mice PI3K activity was immediately suppressed due to the enhanced PTEN activity, whereas genomic ablation of the miR17-92 cluster did not immediately abolish all mature miRNAs of this cluster, which continue to function until degradation based on their turnover rate (51). A similar delayed effect was evident when PI3K activity was enhanced in PTEN-ablated mice (22) relative to P110^*^ Tg mice (Figure 7). Thus, genomic ablation of PTEN does not abolish the function of residual PTEN mRNA and protein in balancing PI3K for development until they are degraded, allowing development up to proB stage. In the P110^*^Tg mice though, we observed a block earlier at the early pro B stage which is driven by the enhanced PI3K activity in these cells.

Interestingly, in mb1-Cre miR17-92^fl/fl^ mice we observed a mild block in B cell development compared to that obtained when miR17-92 is ablated early in hematopoietic progenitors (29), or when the miR17-92 is deleted together with its paralog clusters miR106a-363 and miR106b-25 (41). It is possible that an early deletion of the miR17-92 cluster in the hematopoietic lineage may affect commitment and differentiation into the B lineage, and loss of all paralog clusters aborts all regulatory and compensatory functions by this group of miRNAs. Nevertheless, the block at the proB cell stage that is readily observed in Ventura et al (29), in Nojima et al (52) and here indicates the cell-autonomous important function of the miR17-92 cluster in promoting B cell development at this developmental check-point.

In addition to PTEN, several studies have shown that the miR17-92 controls other important genes regulating the pro-to-preB cell transition such as E2F family of transcription factors (53), as well as the pro-apoptotic Bim (29). Moreover, Lai et al have shown that loss of one PTEN allele fails to rescue B cell development in mice where the miR17-92 is deleted together with its paralog clusters miR106a-363 and miR106b-25 (41). Thus, the miR17-92 cluster and its paralogs are critical for B cell development by regulating multiple target genes controlling developmental transition. The mild pro-to-pre B cell block we show here in miR17-92-deleted or PTENover mice supports this.

Finally, PI3K is most critical for HSCs maintenance and lineage differentiation (54, 55). High level of PI3K (obtained by constitutive active Akt or by PTEN heterozygosity) promotes leukemia and aplastic BM, whereas low level of PI3K prevents self-renewal and differentiation (56, 57). The regulation of PI3K activity in HSCs is unknown. Studies indicate that multipotent HSCs depend on Dicer and miRNA biogenesis for persistence (58), and that PTEN expression regulates HSCs activity by repressing the PI3K/Akt/mTor signaling (59). It is possible that the c-Myc/miR17-92/PTEN axis may also function to regulate PI3K in HSCs. Alternatively, the PTEN-PI3K relationship in balancing PI3K activity may be regulated in the hematopoietic compartment by a different miRNA. Thus, while balancing PI3K is most critical for hematopoiesis in general, the present work proposes that in the B lineage lymphopoiesis PI3K activity is strictly balanced by the c-Myc/miR17-92/PTEN axis to regulate RAG expression and to promote developmental progression.

## Author contributions

DB and DM: conceived of the study; DB, VL, AG, EB, and RD: performed the experiments and analyzed the data. KR, JC, and DM: wrote and edited the manuscript and supervised the experiments.

### Conflict of interest statement

The authors declare that the research was conducted in the absence of any commercial or financial relationships that could be construed as a potential conflict of interest.
