# Supplementary material for: The c-Myc/miR17-92/PTEN Axis Tunes PI3K Activity to Control Expression of Recombination Activating Genes in Early B Cell Development
Source: Front Immunol. 2018 Nov 22;9:2715. doi: 10.3389/fimmu.2018.02715 (PMC6262168; doi:10.3389/fimmu.2018.02715)
Supplement: Supplementary file 1 [file Presentation_1.pdf]

Supplementary information

A.

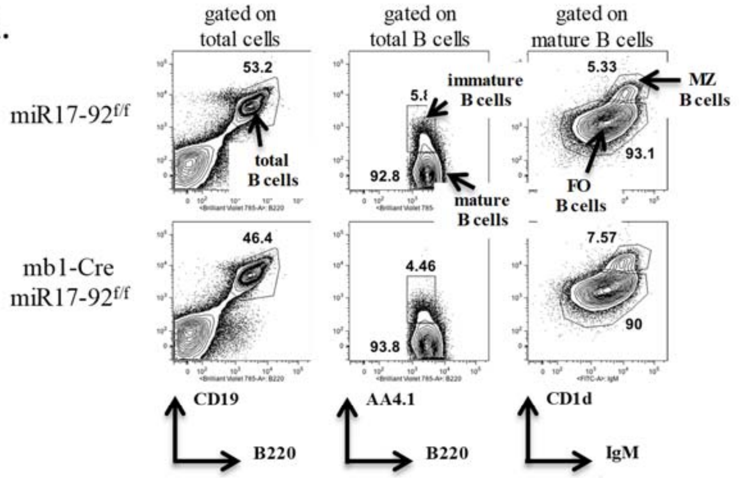

	total B cells (x10^6^)	immature (x10^6^)	mature (x10^6^)	follicular (x10^6^)	MZ (x10^6^)
miR17-92^f/f^	47.96± 3.82	4.10± 0.86	43.44± 4.51	41.05± 4.11	1.63± 0.35
mb1-Cre miR17-92^f/f^	48.97± 3.44	1.96± 0.36	46.80± 3.66	41.26± 2.71	4.23± 0.88

B.

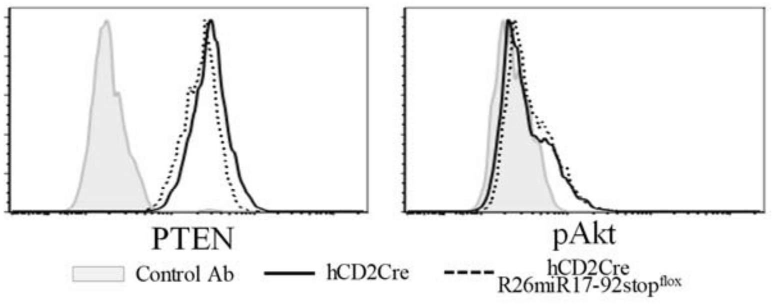

C.

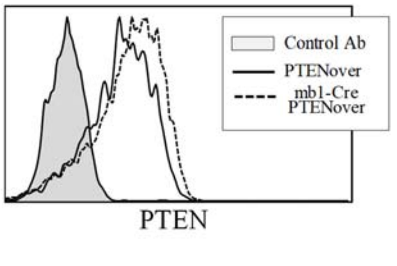

Supplementary figure 1 – (A) - spleen cells from the indicated mice were stained with the indicated antibodies and analyzed by FACS. Gates for the indicated B cell subsets are marked with arrows. Shown are results from representative mice from each group and absolute cell counts.(n=3). (B) - BM cells from the indicated mice were analyzed for intracellular PTEN expression and pAkt by flow cytometry. Analysis was performed on gated B220+/IgM- pro/preB cells. Shown are representative results from 2 mice. (C) - BM from the indicated mice was stained for intracellular PTEN expression and analysis was performed on gated B220+/IgM- pro/preB cells. Shown are representative results from 2 mice.

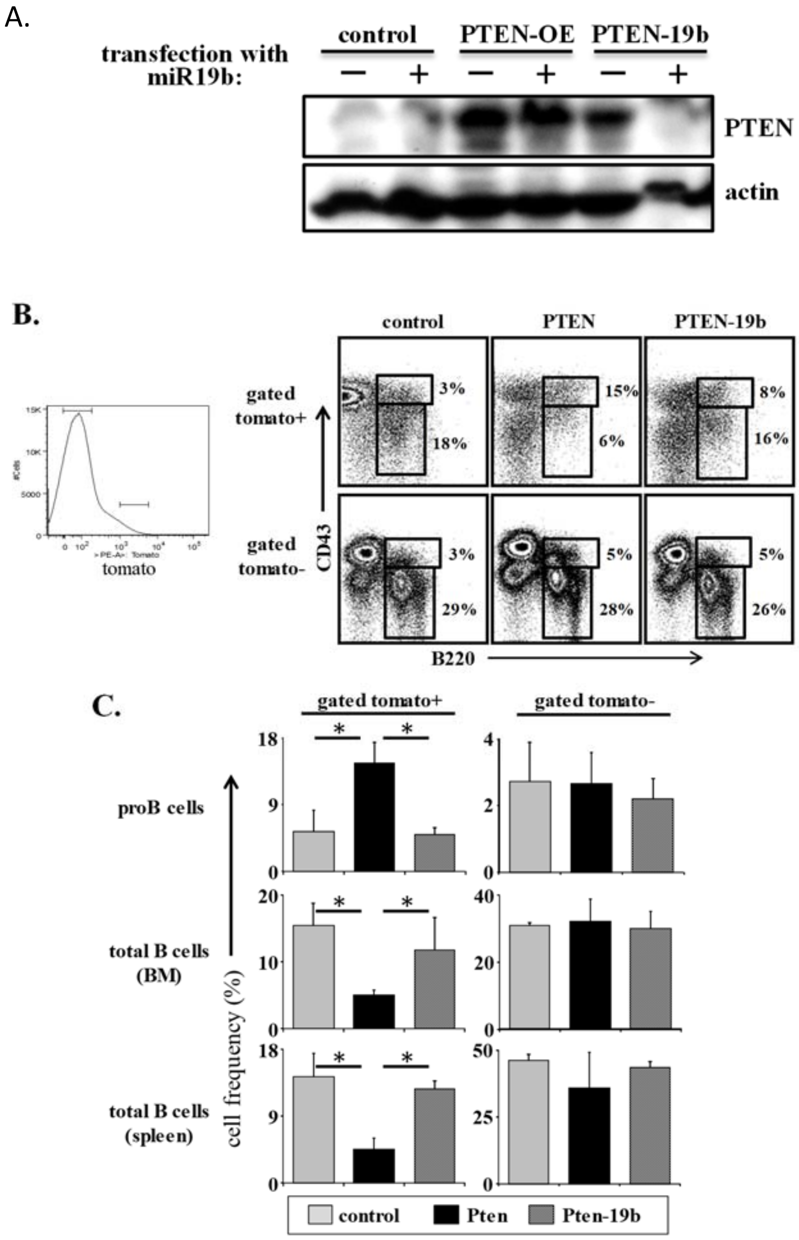

<u>Supplementary figure 2 –</u> (A) - HEK cells were infected with viral vectors encoding human PTEN alone (PTEN-OE) or ligated to a miR19b binding site (PTEN-19b), and an IRES-tomato reporter cassette. Stable clones were selected and subjected to a second transfection with a vector encoding miR19b. Cells were then treated with doxycycline and analyzed for the indicated proteins. (B and C) HSCs from normal mice were transduced with vectors encoding PTEN-OE or PTEN-19B and a tomato reporter cassette (described in A), and used to reconstitute lethally irradiated mice. Briefly, HSCs from WT (CD45.2 C57BL/6) were isolated by FACS sorting for Lineage-negative, cKit-positive, Sca1-positive, CD150-positive and CD48-negative. Primary cells were cultured in Bitarget (Biological industries, Israel), supplemented with 10 ng/ml SCF, TPO, IL-3, and Flt3L (long form, all from Peprotech), together with concentrated Lentiviruses for 48 hours to allow efficient transduction. Viral-transduced cells were transplanted together with congenic competitors CD45.1 (JAX strain 2014) into lethally-irradiated F1 recipients (CD45.1+2). Purification of HSC, and generation of the BM chimeric mice was performed as described in *Bujanover et al Leukemia. 2018 32:2016-2020*. BMs from chimeric mice were analyzed by flow cytometry for the indicated B cell subsets. (B) – representative plots from individual mice. Also shown gates for tomato+ and tomato- cells. (C) – group mean of frequencies of the indicated B cell subsets in BM or spleen. We note that proB cells were defined as B220+/CD43+, which may not be sufficient to accurately detect proB cells.
